# Acetabular cup position differs in spinopelvic mobility types: a prospective observational study of primary total hip arthroplasty patients

**DOI:** 10.1007/s00402-021-04196-1

**Published:** 2021-10-11

**Authors:** Henryk Haffer, Zhen Wang, Zhouyang Hu, Christian Hipfl, Matthias Pumberger

**Affiliations:** Center for Musculoskeletal Surgery, Charité-Universitätsmedizin Berlin, Corporate Member of Freie Universität Berlin, Humboldt-Universität Zu Berlin, and Berlin Institute of Health, Berlin, Germany

**Keywords:** Spinopelvic alignment, Spinal sagittal balance, Total hip arthroplasty dislocation, Total hip replacement, Spinopelvic stiffness, Spinopelvic hypermobility

## Abstract

**Introduction:**

Spinopelvic mobility was identified as a contributing factor for total hip arthroplasty (THA) instability. The influence of spinopelvic function on acetabular cup positioning has not yet been sufficiently investigated in a prospective setting. Therefore, our study aimed (1) to assess cup inclination and anteversion in standing and sitting based on spinopelvic mobility, (2) to identify correlations between cup position and spinopelvic function, (3) and to determine the influence of the individual spinal segments, spinal sagittal balance, and spinopelvic characteristics on the mobility groups.

**Materials and methods:**

A prospective study assessing 197 THA patients was conducted with stereoradiography in standing and sitting position postoperatively. Two independent investigators determined cup anteversion and inclination, C7-Sagittal vertical axis, cervical lordosis (CL), thoracic kyphosis (TK), lumbar lordosis (LL), sacral slope, pelvic tilt (PT), anteinclination (AI), and pelvic femoral angle (PFA). Spinopelvic mobility is defined based on ∆PT = PT_standing_ − PT_sitting_ as ∆PT < 10° stiff, ∆PT ≥ 10–30° normal, and ∆PT > 30° hypermobile. Pearson coefficient represented correlations between the cup position and spinopelvic parameters.

**Results:**

Significant differences were demonstrated for cup anteversion (stiff/hypermobile 29.3°/40.1°; *p* < 0.000) and inclination (stiff/hypermobile 43.5°/60.2°; *p* < 0.000) in sitting, but not in standing position. ∆ (standing/sitting) of the cup anteversion (stiff/neutral/hypermobile 5.8°/12.4°/19.9°; *p* < 0.000) and inclination (stiff/neutral/hypermobile 2.3°/11.2°/18.8°; *p* < 0.000) revealed significant differences between the mobility groups. The acetabular cup position in sitting, was correlated with lumbar flexibility (∆LL) and spinopelvic mobility. Significant differences were detected between the mobility types and acetabular orientation (AI sit:stiff/hypermobile 47.6°/65.4°; *p* < 0.000) and hip motion (∆PFA:stiff/hypermobile 65.8°/37.3°; *p* < 0.000). Assessment of the spinal segments highlighted the role of lumbar flexibility (∆LL:stiff/hypermobile 9.9°/36.2°; *p* < 0.000) in the spinopelvic complex.

**Conclusion:**

The significantly different acetabular cup positions in sitting and in the ∆ between standing and sitting and the significantly altered spinopelvic characteristics in terms of stiff and hypermobile spinopelvic mobility underlined the consideration for preoperative functional radiological assessment. Identifying the patients with altered spinopelvic mechanics due to a standardized screening algorithm is necessary to provide safe acetabular cup positioning. The proximal spinal segments appeared not to be involved in the spinopelvic function.

**Supplementary Information:**

The online version contains supplementary material available at 10.1007/s00402-021-04196-1.

## Introduction

Total hip arthroplasty (THA) is one of the most cost-effective and consistently successful surgeries in orthopaedics; nevertheless, THA instability remains an ongoing challenge [1, 2]. Spinopelvic mobility was identified as a contributing factor for THA dislocation and thus attracted increased attention recently [3–6].

Spinopelvic mobility, as a concept, represents the mutual interaction between spine, pelvis, and hip, enabling the change of different body positions and erect posture [7–9]. Degenerative spine and hip pathologies may alter the spinopelvic biomechanics leading to abnormal spinopelvic mobility [10–12]. Spinopelvic mobility has been described as the physiological change in pelvic tilt from standing to sitting (∆PT). Abnormal spinopelvic mobility is classified as stiff with a change (∆) of PT less than 10° from standing to sitting and a ∆PT of more than 30° as hypermobile [3, 13]. Spinopelvic pathologies are known to contribute to an elevated risk of THA dislocation [11]. Patients with limited pelvic and spinal mobility due to spinal fusion or degeneration have a significantly increased risk of THA dislocation and demonstrated an inferior outcome [14–17]. Furthermore, spinopelvic hypermobility in patients with THA and spinal fusion is also related to a poorer outcome in PROMs and enhanced THA instability [18].

Increased concerns have been raised about Lewinnek Safe Zone (LSZ) for acetabular component positioning in the last years, while investigations have demonstrated a considerable proportion of patients with THA dislocations with components positioned inside the LSZ target values [19–22]. Thus, some authors suggest implant positioning adapted to individual spinopelvic mobility and spinal sagittal balance for instability risk mitigation [23, 24]. In their study, Stefl et al. attempted to align the acetabular component on the basis of the spinopelvic mobility determined preoperatively and to assess the acetabular cup position postoperatively [3]. However, it has not yet been investigated what effect cup positioning without preoperative consideration of spinopelvic mobility has on acetabular cup position in a prospective setting and how individual spinal segments and sagittal spinal balance differ in terms of spinopelvic mobility.

Therefore, our study aimed (1) to assess cup inclination and anteversion in standing and relaxed sitting position in patients after THA based on the spinopelvic mobility, (2) to identify correlations between cup position and spinopelvic parameter, (3) to determine the different spinopelvic characteristics in the mobility groups, (4) and to investigate the influence of the individual spinal segments and spinal sagittal balance on the mobility groups in a holistic approach.

## Materials and methods

### Subjects

A prospective radiological observational study on patients who underwent primary THA at a tertiary referral center between September 2019 and November 2020 was performed. The patients have given their informed written consent, and the investigation is in compliance with the Helsinki Declaration and has been approved by the institutional ethics board (EA2/142/17). THA was performed by four board certified surgeons in supine position via an anterolateral approach aiming for an anatomical acetabular component positioning with target values of 40° inclination and 20° cup anteversion with no technical assistance. The exclusion criteria were any non-elective surgery, bilateral planned THA, severe hip dysplasia with subsequent THA and femur osteotomy, any form of revision THA, ankylosing spondylitis, spinal fusion surgery at any level, osseous metastasis, and neurological pre-existing conditions that significantly influence posture. The prosthesis components and fixation techniques (Supplement Table 1) were selected according to the patient's individual requirements and planned preoperatively using TraumaCad (Brainlab, Munich, Germany). The indications for THA of the included patients were primary osteoarthritis of the hip (*n* = 144), secondary osteoarthritis of the hip divided into the following subgroups dysplasia of the hip (*n* = 21), avascular necrosis of the head (*n* = 14), femoroacetabular impingement type CAM (*n* = 9), and others (*n* = 9).

### Radiographic analysis

**Fig. 1:**
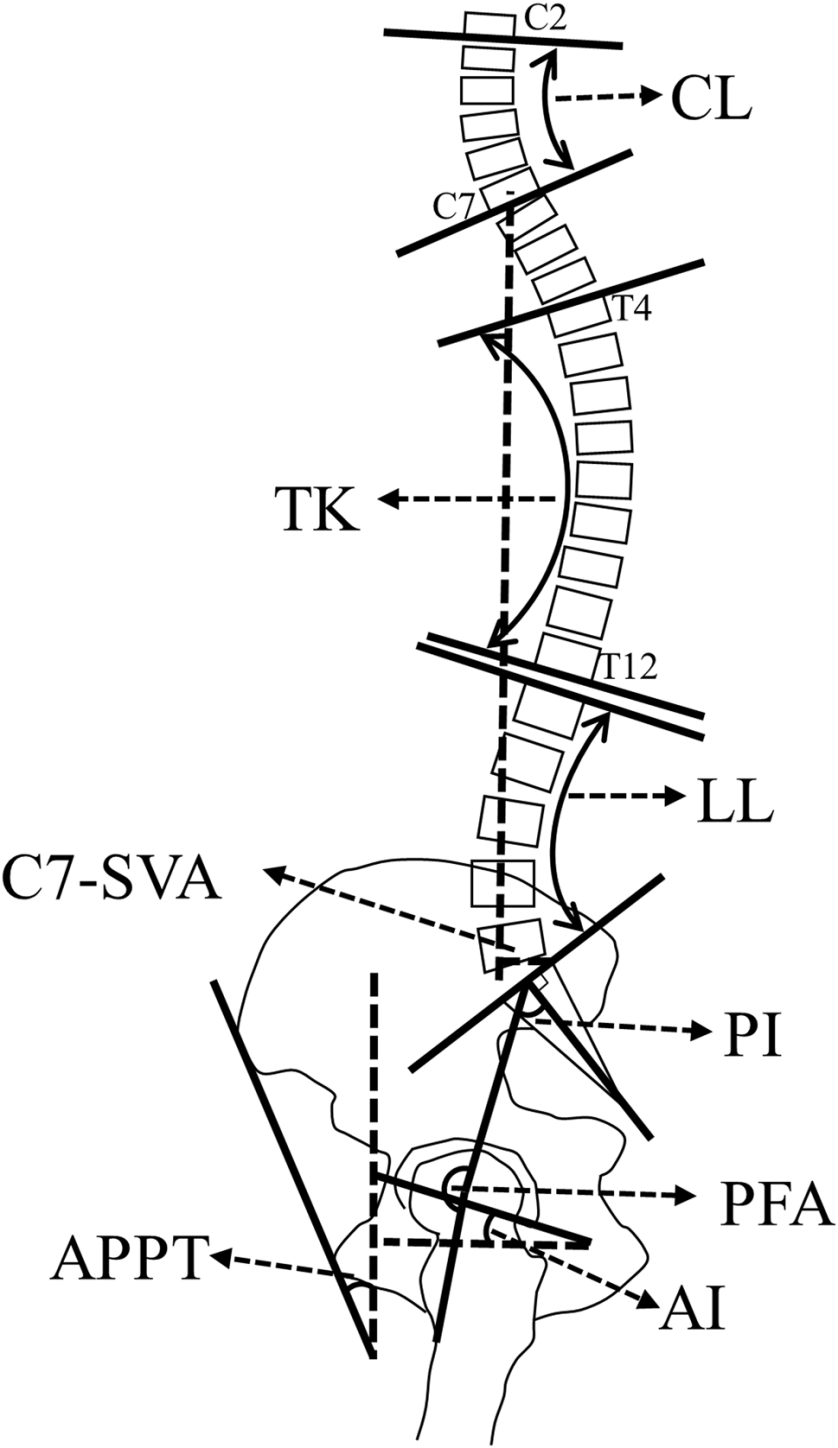
Schematic illustration depicting the measured parameter. *C7-SVA* C7-sagittal vertical axis, *CL* cervical lordosis, *TK* thoracic kyphosis, *LL* lumbar lordosis, *PI* pelvic incidence, *APPT* anterior plane pelvic tilt, *AI* anteinclination, *PFA* pelvic femoral angle

**Fig. 2:**
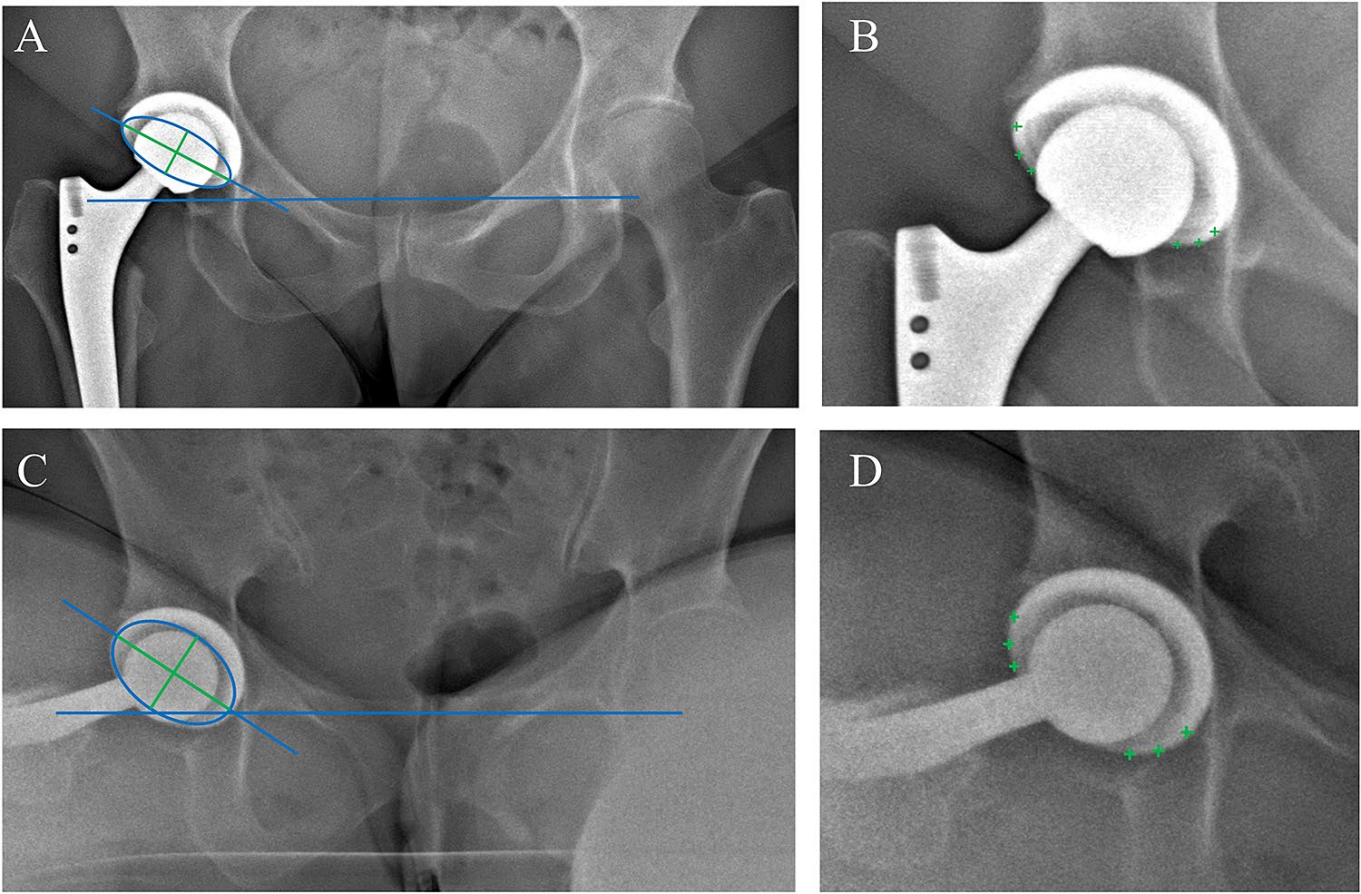
Method of the measurement of acetabular cup anteversion and inclination in a.p. standing (**A**, **B**) and sitting (**C**, **D**) radiographs by defining the rim of the cup (green + in **C**, **D**), and building an ellipse (blue circle; **A**, **C**) with a long and short axis (in green). Blue horizontal line is defined as interteardrop line. Combination of green and blue line displayed the long axis of the ellipse. Anteversion is defined as arc sine (short axis/long axis) of the ellipse and inclination as the angle between interteardrop line and long axis of the ellipse

Within 5–7 days postoperatively, the patients received each a complete spine imaging including the pelvis up to the proximal tibia from lateral and anterior posterior in standing and sitting position using biplanar low-dose stereoradiography (EOS, Paris, France). Patients are advised to stand naturally in the standing position, look forward, and place their hands on a support with relaxed upper limbs, and were instructed to sit relaxed in the seated position on a height-adjustable stool with the femur parallel to the floor. The radiological measurements were conducted by an experienced orthopedic surgeon using Merlin Diagnostic Workcenter (Phoenix PACS, Freiburg, Germany), and a randomly selected 25% dataset was measured by a second independent orthopedic surgeon [25]. Following parameter have been measured postoperatively (Supplement Table 2 for definition and Fig. 1): C7-sagittal vertical axis (C7-SVA), cervical lordosis (CL), thoracic kyphosis (TK), lumbar lordosis (LL), pelvic incidence (PI), sacral slope (SS), pelvic tilt (PT), anterior plane pelvic tilt (APPT), anteinclination (AI), and pelvic femoral angle (PFA). The measurement of cup anteversion and inclination were conducted in standing and sitting anterior posterior radiographs using a reliable method [26]. Inclination was defined as the angle between the line of the long axis of the ellipse and the interteardrop line and anteversion was defined by the trigonometric equation arc sine (short axis/long axis) (Fig. 2). The difference between the standing and sitting values was designated as delta (∆). To classify spinopelvic mobility, we determined ∆PT postoperatively. PT < 10° were defined as stiff, ≥ 10–30° as normal, and > 30° as hypermobile [13].

### Statistical assessment

ANOVA was used to determine differences between the groups regarding spinopelvic mobility. Variance homogeneity was determined by Levene's test. In the case of variance homogeneity, the post hoc analysis according to Bonferroni was applied and in the case of variance inhomogeneity according to Games–Howell. Pearson correlation coefficient was used to represent correlations between parameters. Spearman’s rank correlation coefficient was used to determine the interrater reliability of the radiographic measurements. All statistical analyses were performed using SPSS Version 27 (IBM Corporation, New York, USA). A significance level of *p* < 0.05 was assumed for all tests.

## Results

Three hundred twenty-four patients were screened for study inclusion, and 197 patients were included in the study (all meeting the inclusion and no exclusion criteria). There were 106 female and 91 male patients with a mean age of 66.3 years (17–88 years) and a BMI of 26.8 kg/m^2^ (16.7–51.7 kg/m^2^). Interrater reliability analysis revealed good-to-excellent interobserver agreements (Supplement Table 3) [27].

### Acetabular cup position

**Fig. 3:**
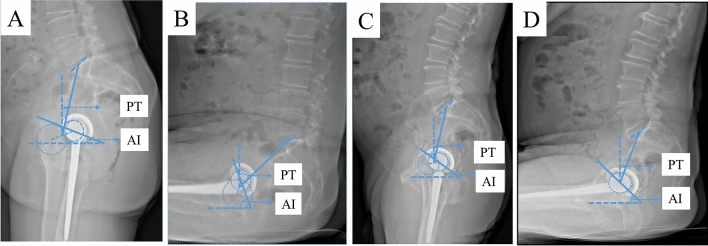
Acetabular cup position in standing and sitting position depicting a patient with spinopelvic hypermobility (**A**, **B**) with standing/sitting PT 11.1°/46.8° resulting in ∆PT 35.7°, AI standing/sitting 20.1°/62.3°. **C**, **D** depicting a patient with spinopelvic stiffness with standing/sitting PT 11.5°/20.8° resulting in ∆PT 9.3°, AI standing/sitting 34.8°/41.2°. Anteinclination (AI), representing as a general combined parameter cup anteversion and inclination, is increased in the patient with spinopelvic hypermobility (AI 62.3°) (**B**) compared to the patient with spinopelvic stiffness (AI 41.2°) (**D**) in sitting position resulting in a more vertical cup position in the hypermobile patient (**B**) and a more horizontal cup position in the stiff patient (**D**)

Acetabular cup position demonstrated significant differences for anteversion and inclination in sitting position between the mobility types (stiff, neutral, and hypermobile) according to the given definition based on ∆PT (Table 1; Fig. 3). No significant difference was found between the types of spinopelvic mobility when measuring cup anteversion and inclination in standing position. Mean values for cup anteversion and inclination in standing position ranged within the established Lewinnek Safe Zone (cup anteversion 15 ± 10° and inclination 40 ± 10°) for all mobility types [21]. Significant deviations in the ∆ from standing to sitting in cup anteversion (cup anteversion standing—cup anteversion sitting: stiff 5.8°; neutral 12.4°; hypermobile 19.9°; all *p* < 0.000) and inclination (cup inclination standing—cup inclination sitting: stiff 2.3°; neutral 11.2°; hypermobile 18.8°; all *p* < 0.000) were detected among the mobility types (Table 2). Particularly noteworthy is the marginal change from standing to sitting in the stiff group in the cup anteversion of 5.8° and inclination of 2.3°. Whereas the cup inclination changed by 18.8° (∆ from standing to sitting) and the cup anteversion by 19.9° in the hypermobile group.

**Table 1:** Acetabular cup position according to postoperative spinopelvic mobility

	Stiff	Neutral	Hypermobile	*p* value^1^	*p* value^2^	*p* value^3^	*η*^2^
Cup anteversion standing° (SD; range)	23.5 (5.9; 10.3–30.9)	24.1 (6.6; 4.7–43.3)	21.7 (7.9; 6.0–41.2)	1.0	0.112	1.0	0.023
Cup inclination standing° (SD; range)	41.2 (8.0; 28.9–56.0)	41.5 (6.2; 25.0–56.7)	41.2 (5.1; 29.7–53.8)	1.0	1.0	1.0	0.001
Cup anteversion sitting° (SD; range)	29.3 (5.9; 17.9–38.7)	36.3 (6.4; 19.0–49.7)	40.1 (5.6; 30.4–51.3)	** < 0.000**	** < 0.000**	** < 0.000**	0.192
Cup inclination sitting° (SD; range)	43.5 (7.6; 29.4–55.1)	52.4 (8.9; 28.2–72.2)	60.2 (9.9; 36.2–79.8)	**0.001**	** < 0.000**	** < 0.000**	0.200

Acetabular cup position in anteversion and inclination in standing and sitting position according to postoperative spinopelvic mobility classification ∆PT. ∆PT < 10° were defined as stiff, ≥ 10–30° as normal, and > 30° as hypermobile

*SD* standard deviation, *η*^2^ ANOVA effect size measure-eta squared

^1^*p* value displayed differences between groups Stiff and Neutral

^2^*p* value between groups Neutral and Hypermobile

^3^*p* value between groups Stiff and Hypermobile. Analysis of Variance (ANOVA) and post hoc analysis according to Bonferroni (due to variance homogeneity) was applied. A significance level of *p* < 0.05 was assumed

**Table 2:** Differences of acetabular cup position between standing and sitting according to postoperative spinopelvic mobility

	Stiff	Neutral	Hypermobile	*p* value^1^	*p* value^2^	*p* value^3^	*η*^2^
∆Cup anteversion standing–sitting ° (SD; range)	− 5.8 (5.5; − 14.7–4.8)	− 12.4 (6.6; − 41.9–7.7)	− 19.9 (6.2; − 32.4–5.0)	** < 0.000**	** < 0.000**	** < 0.000**	0.284
∆Cup inclination standing–sitting ° (SD; range)	− 2.3 (7.2; − 13.1–16.0)	− 11.2 (7.9; − 52.1–15.0)	− 18.8 (8.4; − 36.1–3.5)	** < 0.000**	** < 0.000**	** < 0.000**	0.238

Differences of acetabular cup position in anteversion and inclination between standing and sitting position according to postoperative spinopelvic mobility classification ∆PT. ∆PT < 10° were defined as stiff, ≥ 10–30° as normal and > 30° as hypermobile

*SD* standard deviation. *∆Cup Anteversion Standing–Sitting* difference between cup anteversion in standing position and cup anteversion in sitting position depicted as Delta (∆). *∆Cup Inclination Standing–Sitting* difference between cup inclination in standing position and cup inclination in sitting position depicted as Delta (∆). *η*^*2*^ ANOVA effect size measure-Eta squared

^1^*p* value displayed differences between groups Stiff and Neutral

^2^*p* value between groups Neutral and Hypermobile

^3^*p* value between groups Stiff and Hypermobile. Analysis of Variance (ANOVA) and post hoc analysis according to Bonferroni (due to variance homogeneity) was applied. A significance level of *p* < 0.05 was assumed

### Correlations between spinopelvic characteristics and cup position

**Fig. 4:**
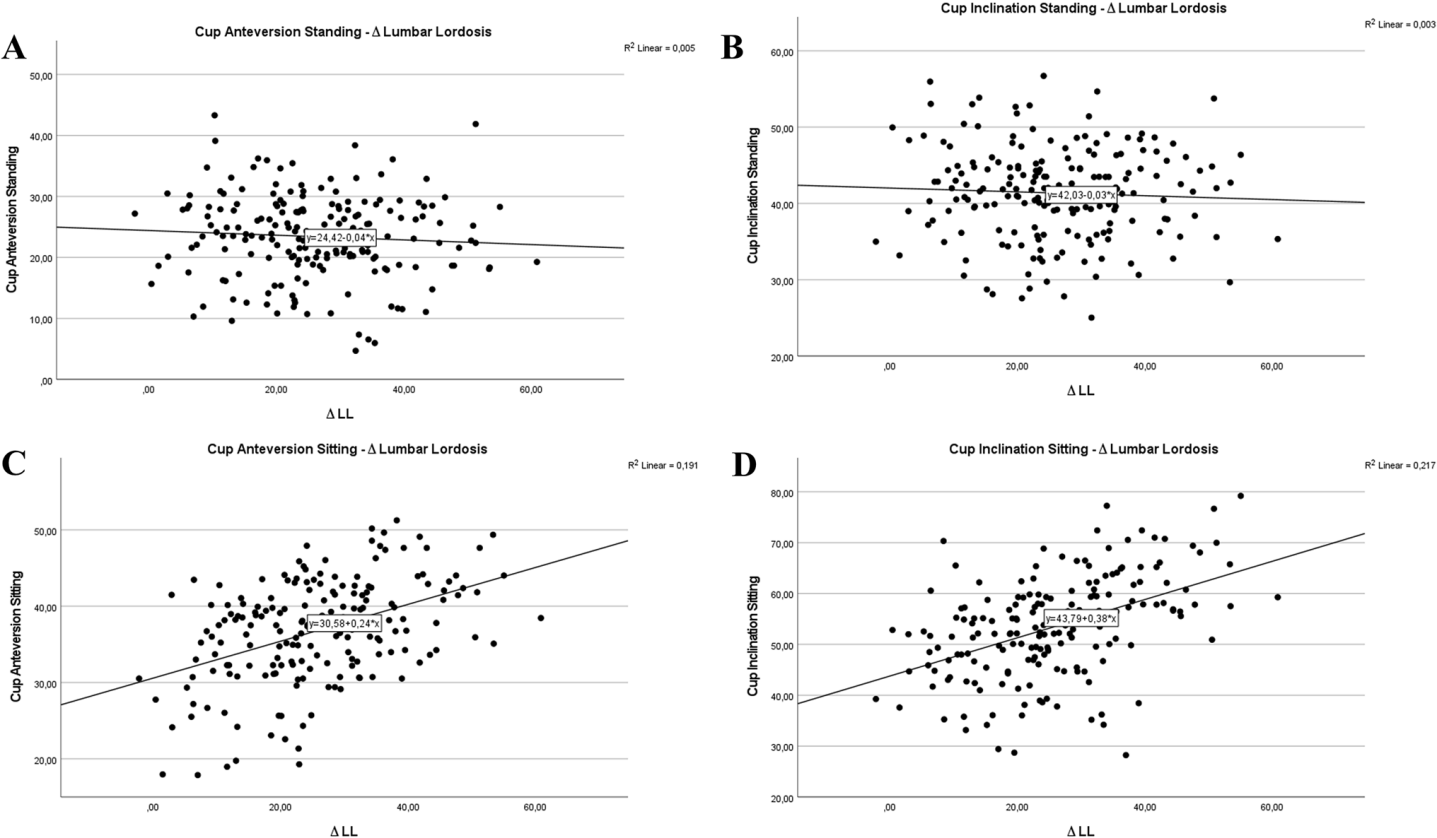
Acetabular cup anteversion and inclination in standing (**A**, **B**) and sitting (**C**, **D**) position related to the postoperative parameter lumbar flexibility represented by ∆LL

**Fig. 5:**
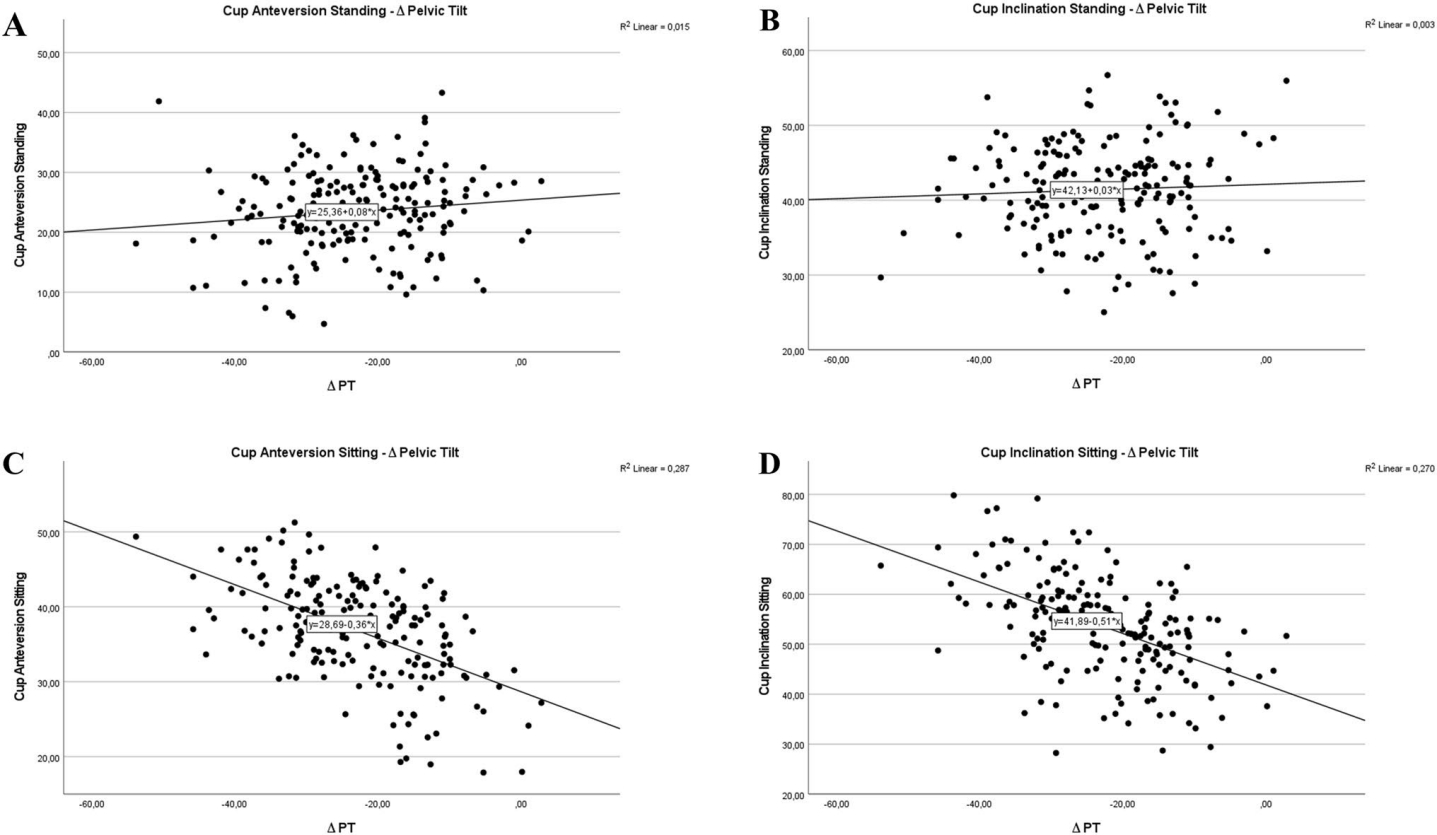
Acetabular cup anteversion and inclination in standing (**A**, **B**) and sitting (**C**, **D**) position related to the postoperative parameter pelvic mobility represented by ∆PT

There were distinct correlations between cup position and spinopelvic characteristics, especially for cup anteversion and inclination in sitting, but not in standing position. These strong correlations were observed between lumbar flexibility (∆ LL) (Fig. 4C and D) and cup position in sitting (cup anteversion sitting: *r* = 0.437; cup inclination sitting *r* = 0.466), but not for standing assessment, demonstrating low correlation of anteversion and inclination in standing to lumbar flexibility (cup anteversion standing: *r* = 0.068; cup inclination standing *r* = 0.051) (Fig. 4A and B). Strong correlations of cup anteversion and inclination in sitting and the pelvic mobility (∆PT) (cup anteversion sitting: *r* = 0.536; cup inclination sitting *r* = 0.520) were demonstrated (Fig. 5C and D), and poor correlation of cup anteversion and inclination in standing (cup anteversion standing: *r* = 0.090; cup inclination standing *r* = 0.054) to pelvic mobility is depicted in Fig. 5A and B.

### Spinopelvic characteristics according to mobility groups

The position of the pelvis reflected in the parameters SS and APPT differs significantly in relation to the spinopelvic mobility types in sitting and in the ∆ from standing to sitting (except APPT in sitting from stiff to neutral group). The similar pattern is apparent in the acetabular orientation represented by AI, where significant differences between the mobility types are found for the sitting and ∆AI values. Hip motion expressed by ∆PFA was significantly different in the three mobility groups, with significantly greater flexibility in the stiff (65.8°) compared to the hypermobile group (37.3°) (Table 3).

**Table 3:** Spinopelvic characteristics according to postoperative spinopelvic mobility

	Stiff	Neutral	Hypermobile	*p* value^1^	*p* value^2^	*p* value^3^
SS stand ° (SD)	39.4 (14.7)	42.7 (8.9)	45.0 (10.8)	0.625	0.516	0.155
SS sit ° (SD)	31.7 (13.0)	22.5 (10.3)	11.3 (11.2)	**0.004**	** < 0.000**	** < 0.000**
∆SS ° (SD)	7.7 (5.4)	20.2 (7.2)	33.7 (8.7)	** < 0.000**	** < 0.000**	** < 0.000**
PFA stand° (SD)	180.2 (8.2)	176.8 (10.0)	169.9 (11.6)	0.615	** < 0.000**	**0.002**
PFA sit° (SD)	114.5 (8.5)	123.7 (12.4)	132.9 (9.6)	**0.009**	** < 0.000**	** < 0.000**
∆PFA° (SD)	65.8 (9.1)	53.1 (10.7)	37.3 (11.6)	** < 0.000**	** < 0.000**	** < 0.000**
AI stand° (SD)	37.8 (9.1)	33.4 (9.1)	31.0 (10.4)	0.242	0.399	**0.041**
AI sit° (SD)	47.6 (10.7)	56.2 (10.5)	65.4 (12.8)	**0.011**	**0.000**	**0.000**
∆ AI° (SD)	− 9.8 (6.1)	− 22.8 (7.6)	− 34.4 (8.3)	** < 0.000**	** < 0.000**	** < 0.000**
APPT stand° (SD)	− 2.0 (6.6)	2.1 (7.2)	7.1 (7.9)	0.106	** < 0.000**	** < 0.000**
APPT sit° (SD)	− 15.0 (6.2)	− 19.2 (9.0)	− 26.4 (11.3)	0.314	** < 0.000**	** < 0.000**
∆APPT° (SD)	13.5 (6.8)	21.3 (8.4)	33.2 (10.0)	0.004	** < 0.000**	** < 0.000**
PI stand° (SD)	57.5 (18.4)	54.3 (12.2)	51.3 (11.7)	1.0	0.484	0.269

Mean spinopelvic parameter in standing, sitting position, and ∆ from standing to sitting according to postoperative spinopelvic mobility classification ∆PT. ∆PT < 10° were defined as stiff, ≥ 10–30° as normal, and > 30° as hypermobile. A significance level of *p* < 0.05 was assumed

*SS* sacral slope, *PFA* pelvic femoral angle, *AI* anteinclination, *APPT* anterior plane pelvic tilt, *PI* pelvic incidence, *Stand* standing position, *Sit* sitting position, *∆* difference from standing to sitting, *SD* standard deviation

^1^*p* value displayed differences between groups Stiff and Neutral

^2^*p* value between groups Neutral and Hypermobile

^3^*p* value between groups Stiff and Hypermobile

### Spinal parameter and sagittal alignment according to the mobility groups and cup position

Spinal sagittal global alignment expressed in C7-SVA standing demonstrated no significant difference between the balance and imbalance group in relation to cup inclination and anteversion in standing and sitting position (anteversion/inclination stand: *p* = 0.256/*p* = 0.188; anteversion/inclination sit: *p* = 0.827/*p* = 0.827). C7-SVA standing revealed no significant differences in relation to the different types of spinopelvic mobility (Table 4). CL and TK in standing, sitting and ∆ from standing to sitting, respectively, displayed no significant differences with respect to the types of spinopelvic mobility. LL in sitting (stiff/ neutral/ hypermobile: 40.1°/28.4°/17.4°) and ∆LL (stiff/ neutral/ hypermobile: 9.9°/24.2°/36.2°) showed significant differences in relation to the various types of spinopelvic mobility.

**Table 4:** Spinal parameter and sagittal spinal balance according to postoperative spinopelvic mobility

	Stiff	Neutral	Hypermobile	*p* value^1^	*p* value^2^	*p* value^3^
CL stand° (SD)	13.0 (11.5)	14.8 (10.2)	16.1 (12.6)	1.0	1.0	1.0
CL Sit° (SD)	14.0 (8.5)	17.4 (11.1)	17.3 (12.2)	0.762	1.0	0.906
∆CL° (SD)	− 1.0 (6.4)	− 2.6 (7.3)	− 1.7 (7.6)	1.0	1.0	1.0
TK Stand° (SD)	38.6 (9.6)	39.7 (12.3)	37.4 (9.3)	1.0	0.706	1.0
TK Sit° (SD)	37.4 (7.5)	37.7 (11.9)	36.8 (11.3)	1.0	1.0	1.0
∆TK° (SD)	1.2 (5.2)	2.0 (5.2)	1.3 (10.1)	1.0	1.0	1.0
LL stand° (SD)	49.9 (18.3)	52.5 (13.4)	52.7 (13.4)	1.0	1.0	1.0
LL sit° (SD)	40.1 (17.8)	28.4 (14.1)	17.4 (13.0)	**0.006**	** < 0.000**	** < 0.000**
∆ LL° (SD)	9.9 (6.8)	24.2 (9.5)	36.2 (12.4)	** < 0.000**	** < 0.000**	** < 0.000**
C7-SVA stand mm (SD)	49.3 (28.3)	54.2 (36.7)	57.7 (34.8)	1.0	1.0	1.0

Mean spinal parameter in standing, sitting position, and ∆ from standing to sitting according to postoperative spinopelvic mobility classification ∆PT. ∆PT < 10° were defined as stiff, ≥ 10–30° as normal and > 30° as hypermobile. A significance level of *p* < 0.05 was assumed

*CL* cervical lordosis, *TK* thoracic kyphosis, *LL* lumbar lordosis, *C7-SVA* C7-sagittal vertical axis, *Stand* standing position, *Sit* sitting position, *∆* difference from standing to sitting, *SD* standard deviation

^1^*p* value displayed differences between groups Stiff and Neutral

^2^*p* value between groups Neutral and Hypermobile

^3^*p* value between groups Stiff and Hypermobile

## Discussion

The investigation of 197 prospectively enrolled patients undergoing total hip replacement with EOS assessment in functional positions demonstrated significant differences for cup anteversion and inclination in sitting, but not in standing position between the types of spinopelvic mobility. It has been illustrated that the acetabular cup position in sitting, but not in standing, was well correlated with the key elements of spinopelvic mobility, namely lumbar flexibility and pelvic mobility. Significant differences were detected between the mobility types and the spinopelvic characteristics in terms of pelvic alignment, acetabular orientation, and hip motion. Examination of the individual spinal elements highlighted the unique role of lumbar function in the spinopelvic complex, compared to the proximal spinal segments and global sagittal spinal alignment.

It was demonstrated that cup anteversion and inclination in sitting position differs significantly between mobility groups. The non-significant differences of the cup position in standing in relation to the mobility types indicated that the shape of spinopelvic mobility is only unmasked in functional examinations. This indicates that the implantation of the acetabular component in a static supine position or the preoperative and postoperative follow-up examinations in a static standing position does not provide sufficient information about possible extreme positions of the pelvis, respectively, and the acetabular component in dynamic situations. Another study assessing spinopelvic mobility reported an acetabular inclination of 40.1° and anteversion of 18.4° in standing position for the normal spinopelvic mobility group, comparable to our results. This illustrates the validity of our data [3].

The minor changes in cup position from standing to sitting in the stiff group may be expected in the presence of limited spinopelvic mobility by definition. The small variations from standing to sitting in cup anteversion (5.8°) and inclination (2.3°) in the stiff group were due to a reduced ability of acetabular opening in sitting position. However, this implied the risk of anterior impingement and posterior THA dislocation during sitting [11]. The substantial alterations from standing to sitting in the hypermobile group regarding the cup position were to be anticipated with great spinopelvic mobility. The changes in cup anteversion of 19.9° reflected the posterior pelvic tilt from standing to sitting. It also confirmed that, as described in the literature, a posterior pelvic tilt is only partially reflected in the increase in cup anteversion (each 1° posterior PT leads to 0.74° acetabular anteversion) [28]. There was a change in cup inclination of 18.8° between standing and sitting in the hypermobile group with a remarkable sitting inclination of more than 60°. Increased acetabular cup inclination is recognized as parameter for an enhanced risk for accelerated surface wear and high ion levels [29, 30].

A close association of the cup position in sitting, but not in standing, with the key elements of spinopelvic function, lumbar flexibility (∆LL), and pelvic mobility (∆PT) was observed. Standing radiographs are standardised, but static. This underlines the need for functional tests to assess the acetabular component position, respectively, and pelvic alignment in different postures. Both standing and sitting positions represent main postures of activities of daily living. Standing radiographs are favorable for detecting posterior THA impingement and sitting radiographs more relevant to anterior impingement most likely.

The same pattern is mainly seen for the spinopelvic characteristics, with significant differences between the mobility types in ∆ and sitting, but not in standing values. In particular, the significant differences of hip motion (∆PFA), a crucial element of spinopelvic function between the stiff (∆PFA 65.8°) and the hypermobile group (∆PFA 37.3°) has to be emphasised. This is considered a clear demonstration of a compensatory mechanism with increased hip flexion, as the pelvic mobility (by definition ∆PT < 10°, but also apparent in ∆ SS (stiff/hypermobile 7.7°/33.7°), is limited. At the same time, the spinal compensation possibility is lacking, since the lumbar flexibility (∆LL) in the stiff group with ∆LL 9.9° is significantly decreased compared with the hypermobile group (∆LL 36.8°). Attempting to understand the mechanisms, another possible explanation might consist in the fact that the patients with limited pelvic mobility do not relieve lumbar lordosis in the sitting position, as physiologically observed, but maintain the lumbar lordosis present in the standing position to enable an upright posture in the sitting position. The second variant could be supported by the observation of non-significantly different LL standing values between the mobility groups. This might suggests not a restriction of lumbar flexibility, but a compensatory mechanism in response to the restricted pelvic mobility. The first hypothesis of reduced lumbar flexibility and limited change in pelvic tilt with overcompensation by hip motion is supported by Esposito et al. [12]. They demonstrated the limited mobility of the spine (decreased ∆LL), not only limited the change in PT, but also lead to an increase in hip motion (∆PFA) in patients who suffered a THA dislocation. They assumed the increased hip motion to be a driving mechanism for anterior impingement and posterior dislocation in sitting position [11]. Supporting this, another study, investigating risk factors for unfavorable pelvic mobility, reported limited lumbar flexibility as associated parameter [31]. Recently, Innmann et al. classified THA patients as “hip users” who have limited lumbar flexibility and compensate for this by increasing hip motion (∆PFA). Identification of “hip users” is recommended by deep-flexed seated position due to a higher accuracy compared to relaxed seated radiographs [32].

The stiff group had significantly more pelvic retroversion in standing (APPT standing stiff/hypermobile − 2°/+ 7.1°), increased acetabular opening (AI standing stiff/hypermobile 37.8°/31.0°), and an enhanced PI in standing (stiff/hypermobile 57.5°/51.3°) than the hypermobile group. When highlighting the contribution of PI, there is evidence that considered abnormal high PI as a risk indicator for THA instability [33]. While, increased pelvic retroversion in standing is reported as an associated factor for unfavorable pelvic mobility and acetabular component orientation [31].

It can be stated that the proximal spinal segments TK and CL do not differ in standing, sitting, or mobility (reflected in ∆) between the spinopelvic mobility groups and are presumably not involved in the adaptive mechanisms of the spinopelvic complex. We were the first to investigate on the global spine and to reveal the proximal spinal segments are not involved in the spinopelvic mobility.

Some limitations of the study need to be addressed. EOS assessments were performed during hospitalization and only short-term follow-up is presented, but long-term follow-up is planned. It might be raised critically that in the initial postoperative period, factors such as pain may have had an influence on the spinopelvic alignment. Therefore, the postoperative pain level of the patients was closely monitored routinely after THA and individually adjusted according to a standardised protocol in cooperation with the anaesthesiological pain service. Despite the short follow-up period, we assume that the postoperative pain level has no relevant influence on the posture of fully mobilised patients with a minimally invasive surgical approach. In our study, the relaxed seated position was selected as the functional assessment and a deep-flexed seated or single leg standing position was not performed as an additional functional exercise. The deep-flexed seated position might be an advantage when identifying “hip users”. These functional images were not possible in the postoperative setting due to patient safety [32, 34, 35]. When evaluating the results, it needs to be considered that the implant positioning was performed in supine position, the spinopelvic assessment analyzed the relaxed seated and standing position, with known strong correlations between standing and supine position [10]. Assessing the risk of THA dislocation, both acetabular component and femoral stem positioning are relevant; in our study, femoral anteversion was not examined. This leads to a potentially incomplete biomechanical representation of the instability risk and should be taken into account as a suspected bias when considering the results. For a better interpretation of the results, we would like to mention that in our patient collective of *n* = 197 patients, *n* = 3 patients were treated with a Smith&Nephew R3 cup and *n* = 2 patients with a ZimmerBiomet TMT cup. The cup position has not been adapted to individual spinopelvic or spinal pathologies, but in the case of the two TMT cups to the acetabular anatomy in patients with progressed secondary osteoarthritis of the hip. It is possible that this may have led to distortions.

In conclusion, significant differences of cup anteversion and inclination were detected in sitting position and in the ∆ between standing and sitting position between the mobility groups. Significant differences in spinopelvic characteristics regarding lumbar flexibility, pelvic alignment, acetabular orientation, and hip motion were demonstrated between the types of mobility providing additional perspectives on mutual interactions in the spinopelvic complex. For the first time, the complete vertebral column, comprising the individual spinal segments, and the sagittal global spinal alignment were depicted in the consideration of the spinopelvic complex in THA patients in a holistic approach. For the preoperative identification of patients with altered spinopelvic mechanics and an associated increased risk of THA instability, a structured medical history (previous spinal surgeries) and physical examination are indispensable. As standard diagnostic, a standing a.p. pelvic X-ray for a better representation of the functional pelvic position is recommended. If there are anamnestic or clinical findings (history of spinal surgery, clinical postural imbalance or advanced arthritic alterations, or surgical changes to the lumbosacral joint) related to spinopelvic mobility or sagittal spinal balance, the radiological diagnostic needs to be extended. For this purpose, standardised lateral radiographs from C7, respectively, and L1 to the femur in standing and sitting position will be conducted. The pelvic tilt can be determined from the lateral standing radiographs (anterior, neutral, or posterior tilt compared to the coronal functional plane). Based on the difference of the pelvic tilt in standing and sitting, the classification of the spinopelvic mobility into stiff (< 10°), normal (≥ 10°–30°), or hypermobile (> 30°) is possible. In addition, the sagittal spinal alignment should be evaluated and a possible sagittal spinal imbalance detected by determining the PI–LL mismatch (pelvic incidence (PI) minus lumbar lordosis (LL); balanced ± 10° or imbalanced > 10°) or the sagittal vertical axis (C7-SVA, pathological > 50 mm). Based on our data, it is recommended to consider individual patient-specific targets based on spinopelvic mobility for acetabular component positioning. Considering our study results with further implications for future research, it can be assumed that increased anteversion for patients with stiff spinopelvic function to avoid anterior impingement and posterior dislocation during sitting is recommended. In patients with hypermobile spinopelvic mobility, we would consider decreased anteversion and inclination; in case of additional sagittal spinal malalignment with pelvic retroversion, this would certainly be contemplated favorable to avoid the risk of posterior impingement with anterior dislocation in standing.

## Supplementary Information

Below is the link to the electronic supplementary material.

Supplementary file1 (TIF 686 kb)

Supplementary file2 (TIF 622 kb)

Supplementary file3 (DOCX 22 kb)

## Data Availability

The datasets generated and analyzed during the current study are available from the corresponding author on reasonable request.
