# Supplementary figures and images for: Acetabular cup position differs in spinopelvic mobility types: a prospective observational study of primary total hip arthroplasty patients
Source: Arch Orthop Trauma Surg. 2021 Oct 11;142(10):2979–89. doi: 10.1007/s00402-021-04196-1 (PMC9474574; doi:10.1007/s00402-021-04196-1)

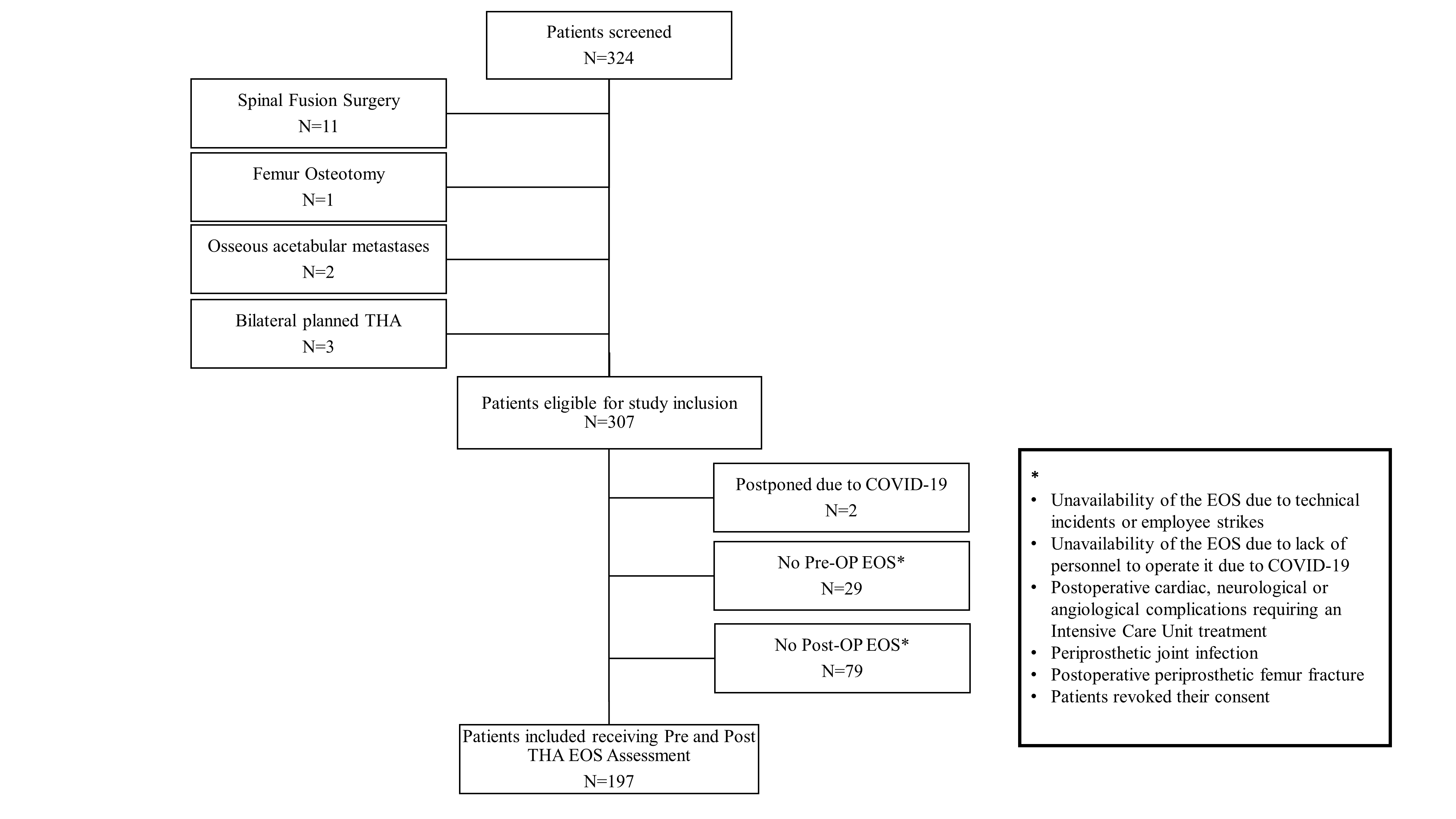

Supplement: Supplementary file 1 — Supplementary file1 (TIF 686 kb) [file 402_2021_4196_MOESM1_ESM.tif]

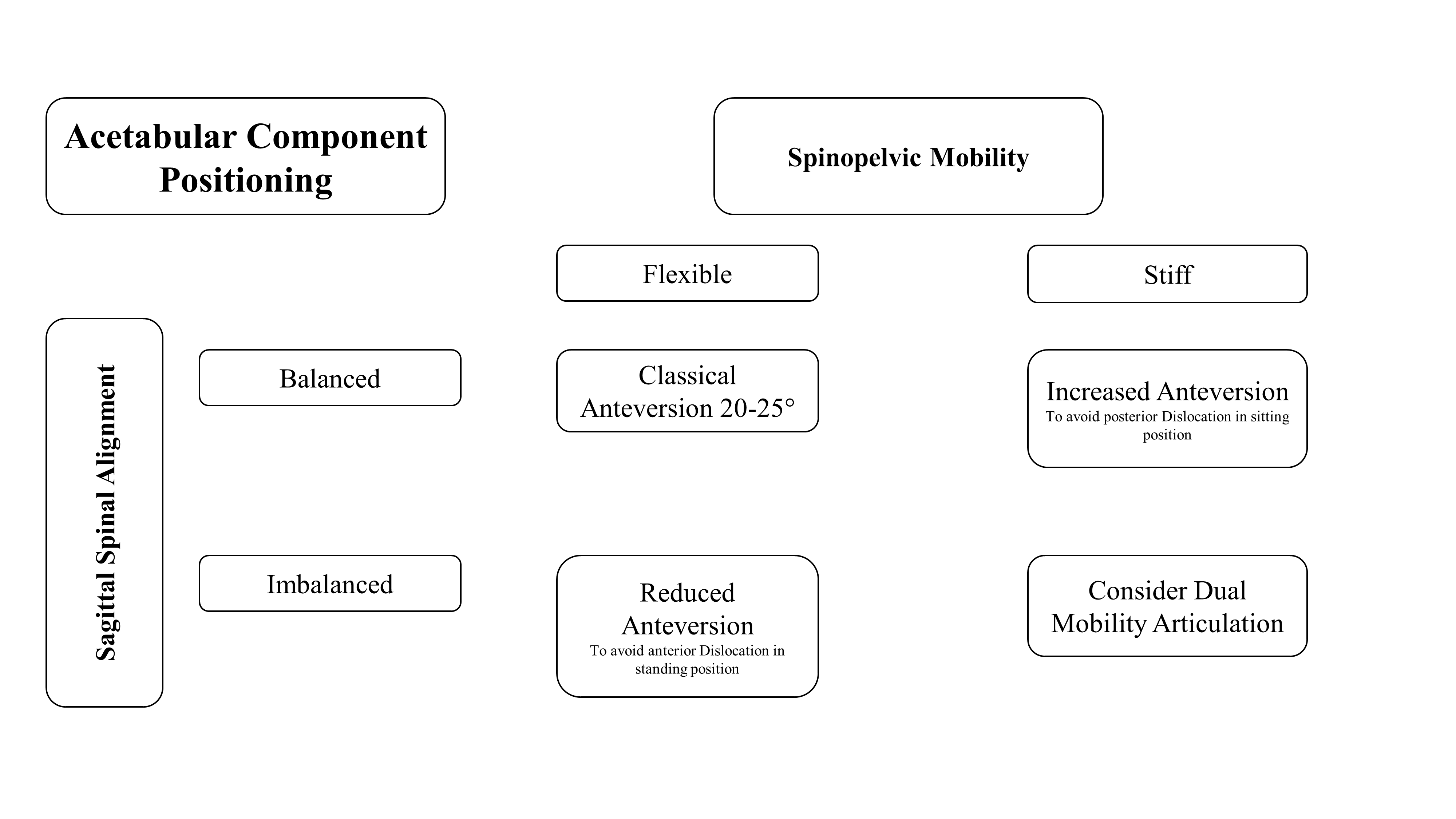

Supplement: Supplementary file 2 — Supplementary file2 (TIF 622 kb) [file 402_2021_4196_MOESM2_ESM.tif]
