# Supplementary material for: Acetabular cup position differs in spinopelvic mobility types: a prospective observational study of primary total hip arthroplasty patients
Source: Arch Orthop Trauma Surg. 2021 Oct 11;142(10):2979–89. doi: 10.1007/s00402-021-04196-1 (PMC9474574; doi:10.1007/s00402-021-04196-1)
Supplement: Supplementary file 3 — Supplementary file3 (DOCX 22 kb) [file 402_2021_4196_MOESM3_ESM.docx]

**Components and Fixation Techniques**

| **Component / Fixation** | **Manufacturer** | **Number (N)** |
| --- | --- | --- |
| Cementless Fixation Technique |  | 197 |
| Allofit Cup | Zimmer Biomet, Warsaw, USA | 192 |
| TMT Cup | Zimmer Biomet, Warsaw, USA | 2 |
| R3 Cup | Smith & Nephew, London, UK | 3 |
| UHMWPE- Inlay | depends on the other components | 194 |
| Ceramic- Inlay | depends on the other components | 3 |
| Ceramic Head | Zimmer Biomet or Smith & Nephew manufactured by Biolox Delta, CeramTec, Plochingen, Germany | 197 |
| SL-Plus MIA Standard Offset Stem | Smith & Nephew, London, UK | 114 |
| SL-Plus MIA Lateral Offset Stem | Smith & Nephew, London, UK | 15 |
| SLR-Plus Stem | Smith & Nephew, London, UK | 1 |
| Avenir Standard Offset Stem | Zimmer Biomet, Warsaw, USA | 14 |
| Avenir Lateral Offset Stem | Zimmer Biomet, Warsaw, USA | 1 |
| Avenir Complete Standard Offset Stem | Zimmer Biomet, Warsaw, USA | 42 |
| Avenir Complete High Offset/  Coxa Vara Stem | Zimmer Biomet, Warsaw, USA | 10 |

**Supplement Table 1.** Overview of the fixation techniques applied the components and manufacturers used.

**Measured Radiological Parameter**

| **Radiological Parameter** | **Description** |
| --- | --- |
| C7-Sagittal vertical axis (C7-SVA) | Horizontal distance between a line from the center of the C7 vertebral body to the posterior superior corner of the sacral endplate and a plumb line from the center of the C7 vertebral body. Assuming >50mm as imbalanced. |
| Cervical lordosis (CL) | Angle between inferior endplate of C2 and inferior endplate of C7 |
| Thoracic kyphosis (TK) | Angle between superior endplate of T4 and inferior endplate T12 |
| Lumbar lordosis (LL) | Angle between superior endplate of L1 and superior endplate of S1 |
| Pelvic incidence (PI) | Angle between the line connecting the midpoint of the superior plate of S1 and the midpoint of the hip axis with the line perpendicular to the superior plate of S1. |
| Sacral slope (SS) | Angle between the superior endplate of S1 and a horizontal line. |
| Pelvic tilt (PT) | Angle between the line joining the midpoint of the hip axis to the midpoint of S1superior endplate and the vertical reference line |
| Anterior plane pelvic tilt (APPT) | Angle between the line connecting the midpoint of both anterior superior iliac spines to the pubic symphysis, and a vertical line. |
| Anteinclination (AI) | combined parameter reflecting the angle from inferior posterior to the anterior superior edge of the acetabulum/ cup and a horizontal line |
| Pelvic femoral angle (PFA) | Angle between the center of the hip axis to the midpoint of the superior sacral endplate and a 10 cm line from the center of the hip axis to the ventral cortex of the femur |

**Supplement Table 2**. Measured radiological parameters with description arranged by global sagittal spinal alignment, sagittal spinal parameter, and spinopelvic parameter.

**Interrater Reliability**

|  |  | **Postoperative** |  | |  |
| --- | --- | --- | --- | --- | --- |
| C7-Sagittal vertical axis |  | .571 | |  | |
| Cervical lordosis |  | .691 | |  | |
| Thoracic kyphosis |  | .638 | |  | |
| Lumbar Lordosis |  | .779 | |  | |
| Pelvic incidence |  | .728 | |  | |
| Sacral slope |  | .711 | |  | |
| Pelvic tilt |  | .816 | |  | |
| Anterior plane pelvic tilt |  | .554 | |  | |
| Anteinclination |  | .743 | |  | |
| Pelvic femoral angle |  | .831 | |  | |

**Supplement Table 3.** Postoperative values of interrater reliability depicting the global spinal alignment, spinal sagittal and spinopelvic parameter. Spearman´s rank correlation coefficient was used.

**Acetabular Cup Position according to the Gender**

|  | Female  (n=106) | Male  (n=91) | P-value |
| --- | --- | --- | --- |
| Cup Anteversion Standing ° (SD; range) | 24.3 (7.1; 6.0-41.9) | 22.4 (6.7; 4.7-43.3) | 0.061 |
| Cup Inclination Standing ° (SD; range) | 40.7 (6.6; 25.0-56.0) | 42.1 (5.5; 29.7-56.7) | 0.113 |
| Cup Anteversion Sitting ° (SD; range) | 36.8 (7.0; 17.9-51.3) | 36.7 (6.6; 18.0-49.4) | 0.918 |
| Cup Inclination Sitting ° (SD; range) | 52.9 (10.4; 28.7-79.2) | 54.3 (9.8; 28.2-79.8) | 0.354 |

**Supplement Table 4.** Acetabular cup position in anteversion and inclination in standing and sitting position according to the Gender. P-value displayed differences between groups female and male. SD=standard deviation. Unpaired T-Test was used.

**Spinopelvic Characteristics according to the Gender**

|  | Female  (n=106) | Male  (n=91) | P-value |
| --- | --- | --- | --- |
| SS Stand ° (SD) | 44.6 (9.4) | 41.1 (10.4) | **0.014** |
| SS Sit ° (SD) | 22.4 (11.4) | 18.4 (12.8) | **0.022** |
| ∆ SS ° (SD) | 22.3 (10.3) | 22.7 (10.6) | 0.776 |
| PFA Stand ° (SD) | 175.5 (11.9) | 175.2 (9.3) | 0.854 |
| PFA Sit ° (SD) | 126.2 (11.0) | 123.9 (14.1) | 0.216 |
| ∆ PFA ° (SD) | 49.4 (12.7) | 51.2 (14.4) | 0.355 |
| AI Stand ° (SD) | 35.1 (9.6) | 31.1 (9.1) | **0.003** |
| AI Sit ° (SD) | 59.1 (12.2) | 56.2 (11.9) | 0.098 |
| ∆ AI ° (SD) | -24.1 (9.9) | -25.2 (10.3) | 0.456 |
| APPT Stand ° (SD) | 4.2 (7.8) | 1.5 (7.2) | **0.015** |
| APPT Sit ° (SD) | -18.8 (10.2) | -22.7 (9.4) | **0.006** |
| ∆ APPT ° (SD) | 22.9 (10.7) | 24.3 (10.2) | 0.346 |
| PI Stand ° (SD) | 55.8 (13.4) | 51.5 (11.4) | **0.018** |

**Supplement Table 5.** Mean spinopelvic parameter in standing, sitting position and ∆ from standing to sitting according to the gender. P-Value displayed differences between gender groups female and male. SS=sacral slope; PFA=pelvic femoral angle; AI=anteinclination; APPT=anterior plane pelvic tilt; PI=pelvic incidence; Stand=standing position; Sit=sitting position; ∆=difference from standing to sitting; SD=standard deviation. Significant differences are marked in bold. Unpaired T-Test was used.

**Spinal Parameter and Sagittal Spinal Balance according to the Gender**

|  | Female  (n=106) | Male  (n=91) | P-value |
| --- | --- | --- | --- |
| CL Stand ° (SD) | 13.0 (10.1) | 17.4 (11.4) | **0.005** |
| CL Sit ° (SD) | 15.2 (11.7) | 19.3 (10.2) | **0.012** |
| ∆ CL ° (SD) | -2.4 (7.5) | -2.1 (7.0) | 0.805 |
| TK Stand ° (SD) | 37.6 (11.5) | 40.6 (11.1) | 0.064 |
| TK Sit ° (SD) | 36.2 (11.9) | 38.9 (10.7) | 0.113 |
| ∆ TK ° (SD) | 1.8 (7.2) | 1.8 (6.1) | 0.974 |
| LL Stand ° (SD) | 54.5 (11.2) | 49.9 (15.1) | **0.023** |
| LL Sit ° (SD) | 28.8 (15.5) | 24.3 (15.0) | **0.040** |
| ∆ LL ° (SD) | 25.9 (12.4) | 25.9 (12.1) | 0.966 |
| C7-SVA stand mm (SD) | 49.3 (33.4) | 60.8 (35.5) | **0.021** |

**Supplement Table 6.** Mean spinal parameter in standing, sitting position and ∆ from standing to sitting according to the gender. P-value displayed differences between gender groups female and male. CL=cervical lordosis; TK=thoracic kyphosis; LL=lumbar lordosis; C7-SVA=C7-sagittal vertical axis; Stand=standing position; Sit=sitting position; ∆=difference from standing to sitting: SD=standard deviation. Significant differences are marked in bold. Unpaired T-Test was used.
